# The development of white matter structural changes during the process of deterioration of the visual field

**DOI:** 10.1038/s41598-018-38430-5

**Published:** 2019-02-14

**Authors:** Shir Hofstetter, Norman Sabbah, Saddek Mohand-Saïd, José-Alain Sahel, Christophe Habas, Avinoam B. Safran, Amir Amedi

**Affiliations:** 1 Department of Medical Neurobiology, The Institute for Medical Research Israel–Canada, Faculty of Medicine, The Hebrew University of Jerusalem, 91220 Jerusalem, Israel; 2 The Edmond and Lily Safra Center for Brain Sciences, The Hebrew University of Jerusalem, 91220 Jerusalem, Israel; 3 Sorbonne Université, INSERM, CNRS, Institut de la Vision, F-75012 Paris, France; 4 CHNO des Quinze-Vingts, DHU Sight Restore, INSERM-DGOS CIC 1423, F-75012 Paris, France; 5 Fondation Ophtalmologique A. de Rothschild, F-75019 Paris, France; 6 Department of Ophthalmology, University of Pittsburgh School of Medicine, Pittsburgh, PA 15213 USA; 7 Department of Clinical Neurosciences, Geneva University School of Medicine, Geneva, Switzerland; 8 Centre de Neuro-Imagerie, Centre Hospitalier National d’Ophtalmologie des Quinze-Vingts, Paris, F-75012 France; 9 The Cognitive Science Program, The Hebrew University of Jerusalem, 91220 Jerusalem, Israel

## Abstract

Emerging evidence suggests that white matter plasticity in the adult brain is preserved after sensory and behavioral modifications. However, little is known about the progression of structural changes during the process of decline in visual input. Here we studied two groups of patients suffering from advanced retinitis pigmentosa with specific deterioration of the visual field: patients who had lost their peripheral visual field, retaining only central (“tunnel”) vision, and blind patients with complete visual field loss. Testing of these homogeneous groups made it possible to assess the extent to which the white matter is affected by loss of partial visual input and whether partially preserved visual input suffices to sustain stability in tracts beyond the primary visual system. Our results showed gradual changes in diffusivity that are indicative of degenerative processes in the primary visual pathway comprising the optic tract and the optic radiation. Interestingly, changes were also found in tracts of the ventral stream and the corticospinal fasciculus, depicting a gradual reorganisation of these tracts consequentially to the gradual loss of visual field coverage (from intact perception to partial vision to complete blindness). This reorganisation may point to microstructural plasticity underlying adaptive behavior and cross-modal integration after partial visual deprivation.

## Introduction

One of the most intriguing and important characteristics of the brain is its lifelong ability to modify and adapt, not only as a consequence of pathology, but also in response to behavioral and environmental adaptations. The neural components of the gray and white matter undergo both functional and structural modifications in a highly dynamic time scale^1^. However, the capacity for brain reorganisation varies across different sensory and cognitive modalities, and some neural functions demonstrate little or no ability to adapt after critical periods in development^2–4^. The functional and structural impact of visual deprivation has often been studied in congenitally or early blind subjects, both for clinical purposes and as a model of brain plasticity (reviewed in^5,6^). In recent years such research has been extended to study the effects of visual loss in adulthood. Although the extent of plasticity is generally thought to decrease after childhood, studies of subjects who lost their vision in adulthood indicate that the nervous system does preserve its capacity for modification^6^. Specifically, changes in the microstructure of white matter tracts have been found in late blind subjects when compared to the congenitally blind^7^ or to sighted controls^8–10^. These studies commonly point to alterations in the optic radiation (OR)^7–9^. A number of studies have explored the ways in which this primary afferent pathway is impacted by the pathological effects of ophthalmic diseases that obscure the visual field but do not cause complete blindness^11–14^.

Beyond those alterations in the primary afferent tracts conveying direct visual input from the retina to the visual cortex, a recent study of congenitally and late blind subjects found microstructural changes in association fasciculi involved in visual processing also in the ventral visual stream^10^, suggesting that visual deprivation in adulthood has a more extensive impact on neuronal networks. A few studies have also investigated non-visual tracts, such as the corticospinal tract (CST)^7–9^. The changes that we found in those studies, however, were inconsistent.

Retinitis pigmentosa (RP) is an inherited retinal disease in which degeneration of the photoreceptors induces a characteristic visual field deficit. During the progressive phase of the disease the peripheral visual field deteriorates leaving the central vision intact, giving rise to “tunnel vision”. Eventually the disease leads to complete blindness^15,16^, although some patients still retain weak perception of light^16^. A recent study by Sabbah *et al*. (including most of the current authors) revealed gradual changes in functional connectivity between the language areas and the occipital cortex in RP patients as the disease progressed from partial to complete visual loss^17^. This raised the intriguing question of whether a similar ongoing pattern of change would be observed in the structural properties of the underlying white matter.

Diffusion tensor imaging (DTI) is a well-established method for the study of white matter microstructure *in vivo*. Owing to its non-invasive nature and sensitivity to the underlying microstructure, DTI has been used in numerous studies to investigate white matter degeneration following ophthalmological diseases or injuries^12–14,18–21^. Here we used the DTI framework to study the progression of white matter plasticity in a state of deteriorating visual field perception in a group of RP patients with preserved central visual field (“tunnel vision”), compared to that in blind RP patients (i.e., with no retention of visual acuity) and in age-matched sighted controls. Studying these highly specific groups with homogeneous retinal deficiencies made it possible to examine the extent to which white matter plasticity is affected by partial loss of visual input. Specifically, we examined whether the partially preserved visual input was enough to sustain stability versus plasticity in tracts beyond the primary visual system, as suggested by a recent study in patients with central scotoma^14^, or whether the lack of peripheral visual input initiates structural changes that progress as vision deteriorates. Overall, we evaluated the effects, if any, of preserved partial visual input on the structure of main white matter fasciculi compared to matched healthy controls to blind patients in the final stages of the disease, i.e., with no retained visual input.

Based on previous findings of white matter structural plasticity following late blindness, here we examined the tracts of the visual system, but we also extended the study focus to the main tract of the motor system (the CST)^7–10^, for which previous studies have reported mixed results. Within the visual system we focused on the primary afferent visual pathway (optic tract and optic radiation) sending visual input to the visual cortex, and two associated tracts that comprise the visual ventral stream (the inferior fronto-occipital fasciculus (IFOF), which connects the occipital and the frontal cortices, and the inferior longitudinal fasciculus (ILF), which conveys visual information from the occipital cortex to the temporal lobe^22,23^). Also evaluated in these tracts were correlation analyses between diffusion indices and physiological measures of visual acuity and visual deficit duration. Changes in the underlying microstructure of these tracts as vision deteriorates from sight to partial blindness to complete blindness were analysed statistically using tract-based spatial statistics (TBSS)^24^.

## Results

### Structural changes in the optic pathway

**Figure 1:**
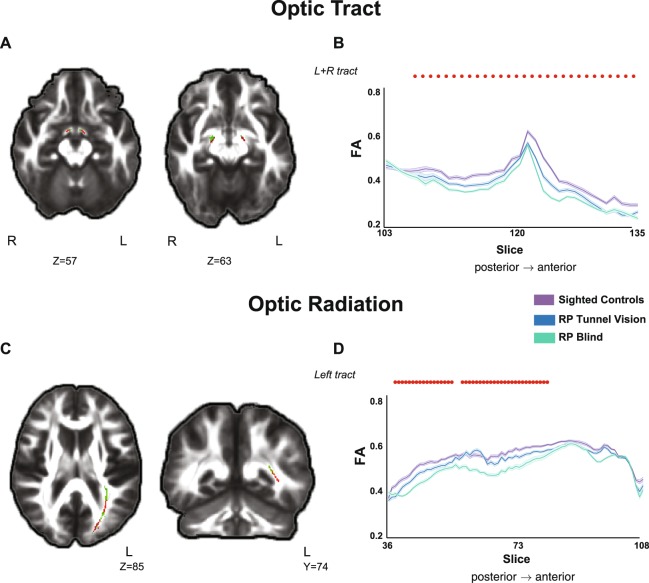
Gradual changes in diffusivity in the visual pathway following deterioration of the visual field in patients suffering from retinitis pigmentosa (RP). (**A**) Statistical map (red voxels) presents the linear trend of reduction in FA values in the optic tract (green voxels) from sighted subjects to RP-TV subjects to RP-BL subjects. (**B**) FA values along the optic tract mask are presented for the three groups. Bold contours correspond to the mean of each group, and the light shaded areas show ±1 SEM. Red dots represent the location of significant voxels that were found in the statistical analysis (p < 0.05, corrected). (**C**) TBSS statistical map (red voxels) presents a linear trend of reduction in FA values in the optic radiation (green voxels). (**D**) FA values along the optic radiation mask are presented for the three groups. Bold contours correspond to the mean of each group, and the light shaded areas show ± 1 SEM. Red dots represent the location of significant voxels that were found in the statistical analysis (p < 0.05, corrected). TBSS-analysed statistical maps are overlaid on the mean FA image and the study-specific skeleton mask of the visual pathway (optic tract or optic radiation, green voxels) at a threshold of p < 0.05 (corrected). R and L indicates the right and left side of the brain, respectively. Z corresponds to Z views in MNI coordinates. For demonstrative purpose the FA values along the tract masks were averaged for each group within in each slice.

Within the optic pathway we found a linear trend of change in fractional anisotropy (FA), corresponding to the level of (residual) visual input accompanying the progression of the visual disease. In other words, we found clusters in which FA values diminished progressively from sighted controls to RP patients with tunnel vision (RP-TV) to RP patients with no visual perception (RP-BL) (p < 0.05, corrected). Significant clusters were found in the optic radiation, as well as in the optic tract, close to the optic chiasma (Fig. 1). To confirm that the clusters found in the optic tract in our TBSS analysis were indeed located within this specific white matter tract in all subjects, we de-projected the significant cluster to each subject’s native space. The results showed an opposite trend, namely in radial diffusivity (RD) values in the optic radiation, with gradually increasing values from sighted controls to RP-TV subjects to RP-BL individuals (p < 0.05, corrected).

There were no other significant gradual changes in the optic pathway for the other DTI indices (mean diffusivity (MD) and axial diffusivity (AD)).

### Structural changes in the ventral stream

**Figure 2:**
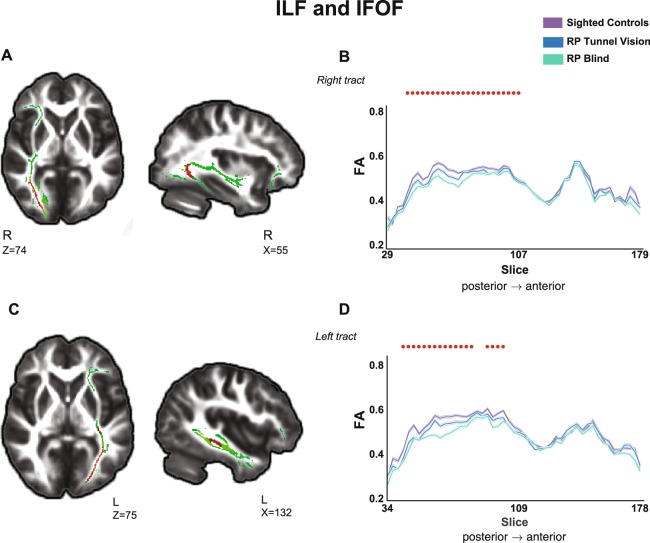
Gradual changes in diffusivity in the visual ventral stream. (**A**) TBSS statistical maps (red voxels) present a significant linear trend of reduction in FA values in the right inferior longitudinal fasciculus (ILF) and inferior fronto-occipital fasciculus (IFOF) (green voxels) from sighted subjects to RP-TV subjects to RP-BL subjects. (**B**). FA values along the right ILF and IFOF mask are presented for the three groups. Bold contours correspond to the mean of each group, and the light shaded areas show ±1 SEM. Red dots represent the location of significant voxels that were found in the statistical analysis (p < 0.05, corrected). (**C**) Statistical maps (red voxels) present a significant linear trend of reduction in FA values in the left ILF and IFOF (green voxels) from sighted subjects to RP-TV subjects to RP-BL subjects. (**D**) FA values along the left ILF and IFOF mask are presented for the three groups. Bold contours correspond to the mean of each group, and the light shaded areas show ±1 SEM. Red dots represent the location of significant voxels that were found in the statistical analysis (p < 0.05, corrected). TBSS-analysed statistical maps are overlaid on the mean FA image and the study-specific skeleton mask of the ventral pathway (ILF and IFOF, green voxels) at a threshold of p < 0.05 (corrected). R and L indicates the right and left side of the brain, respectively. Z and X correspond to Z and X views in MNI coordinates. For demonstrative purpose the FA values along the tract masks were averaged for each group within in each slice and presented using a sampling factor of 3.

We also observed a gradual reduction in FA from sighted controls to RP-TV patients to blind RP-BL patients in the posterior parts of the ILF and IFOF (see Fig. 2). As in the optic pathway, radial diffusivity values showed a tendency to increase over the three groups (p < 0.05, corrected). There were no significant changes in mean diffusivity or in axial diffusivity in this ventral stream.

### Changes in the corticospinal tract

**Figure 3:**
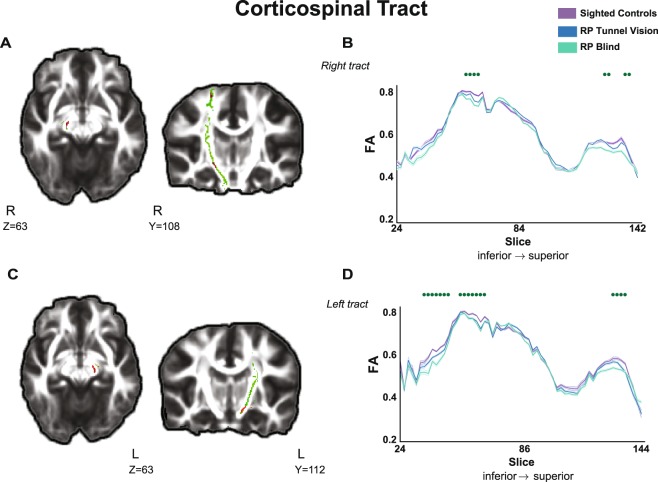
Gradual changes in diffusivity in the corticospinal tract (CST). (**A**) TBSS-analysed statistical map (red voxels) presents the linear trend of reduction in FA values from sighted subjects to RP-TV subjects to RP-BL subjects in the right CST (green voxels) passing through the brainstem. (**B**) FA values along the right CST mask are presented for the three groups. Bold contours correspond to the mean of each group, and the light shaded areas show ±1 SEM. Green dots represent the location of significant voxels that were found in the statistical analysis (p < 0.005, not corrected). (**C**) Statistical map (red voxels) presents a similar linear trend of reduction in FA values from sighted subjects to RP-TV subjects to RP-BL subjects in the left CST (green voxels). (**D**) FA values along the right CST mask are presented for the three groups. Bold contours correspond to the mean of each group, and the light shaded areas show ±1 SEM. Green dots represent the location of significant voxels that were found in the statistical analysis (p < 0.005, not corrected). TBSS-analysed statistical maps are overlaid on the mean FA image and the study-specific skeleton mask of the CST (green voxels) at a threshold of p < 0.01 (for illustration purposes). R and L indicates the right and left side of the brain, respectively. Z and Y correspond to Z and Y views in MNI coordinates. For demonstrative purpose the FA values along the tract masks were averaged for each group within in each slice and presented using a sampling factor of 2.

We found a significant trend of change along the corticospinal tract only when we used the less stringent threshold of p < 0.005 (uncorrected for multiple comparisons). A similar trend of FA reduction with diminishing visual field was observed in the route of the tracts through the brainstem, from the crus cerebri to the pyramids (see Fig. 3). Progressive reduction in axial diffusivity appeared in the bilateral corticospinal tract in similar clusters along the brainstem. In the right corticospinal tract, changes were also evident near the somatosensory cortex. No changes in radial diffusivity or mean diffusivity were found at this threshold.

### Correlations with physiological measures

In the RP-TV group we performed partial correlation analyses between diffusion indices, visual acuity and visual deficiency duration, while controlling for age. TBSS revealed significant positive correlations (p < 0.05, corrected) between visual acuity scores and radial diffusivity values in the right optic radiation, and a similar correlation between visual acuity scores and mean diffusivity values. When controlling for visual acuity scores and age, no correlation was found in this group between diffusion indices and the duration of visual deficiency. These results support an effect of diminishing visual acuity on the microstructure of the primary optic pathway.

## Discussion

In this study we explored the effects of preserved partial vision on the microstructure of prominent white matter tracts. Using a unique homogeneous group of RP patients with retained central tunnel vision, we documented the gradual changes that we observed in DTI indices as the degenerative RP condition progressed to complete loss of visual perception, and which suggested that some reorganisation of the white matter tracts takes place even when some vision remains in parts of the visual field. Analyses of the visual pathway from the optic chiasma to the primary visual cortex (optic tract and optic radiation) revealed a gradual decline in diffusion anisotropy (FA), driven by an increase in radial diffusivity. In tracts not directly linked to input deficiency, such as the association tracts of the ventral visual stream (the ILF and the IFOF) and the corticospinal tract, a similar pattern of gradually decreasing FA also emerged.

We also observed a decrease in diffusion anisotropy in the visual pathways both anterior and posterior to the relay station in the lateral geniculate body. In the optic tract, reduced FA values were found in a cluster near the optic chiasma. A few studies have described the effects of complete blindness in adulthood on the retino-thalamic tract. Zhang *et al*. reported reduced FA and axial diffusivity, together with increased radial diffusivity, and mean diffusivity, in the optic nerves of patients with acquired blindness^25^. In previous studies of ophthalmological diseases in which the visual field was partially darkened, reduced FA was reported in the optic nerve and tract^14^, and the state of disease and severity of visual field defects were found to be correlated with FA values^11,12,21,26^. In the optic radiation, reduced FA values were reported in groups of congenitally or early blind individuals^7,9,27,28^, but were not found in late blind subjects. Whereas in certain studies no significant changes were found in the optic radiation when blindness was acquired late in life^25^, more recent studies have reported reduced FA values in the optic radiation^7–9^. Our finding of reduced FA and increased radial diffusivity values in the optic radiation, as well as the correlation we observed between changes in mean diffusivity and visual acuity scores, are consistent with those DTI studies, and reveal a gradual progression of structural change indicative of a degenerative process through the diminishing of visual input.

Studies documenting cross-modal plasticity in the blind have posited that the occipital cortex may be recruited for other modes of sensory and cognitive processing such as memory and language^29–40^. We found a gradual change in the microstructure of two related ventral white matter networks: the IFOF, which connects the occipital and frontal lobes directly^22^ and is thought to be involved in reading and writing as well as in language semantics^22,41,42^, and the inferior longitudinal fasciculus (ILF), which connects the occipital with temporal lobes and is related to visual memory, visual perception and language processes such as reading and semantic processing^22,23^. Changes in diffusivity of reduced FA and increased radial diffusivity have been reported in these tracts in groups of congenitally and late blind individuals^8–10^. In the present study we did not examine a direct relationship between microstructural changes in these tracts and behavioral measures that might indicate adaptation to linguistic abilities. However, a functional analysis at resting state conducted in the same three groups of subjects did indicate gradually increasing functional connectivity between Broca’s area and the primary visual areas^17^, supporting the supposition that language-related cross-modal plasticity can indeed be achieved in the late blind. Interestingly, intermediate connectivity (between negative and positive functional correlations observed in the sighted and the RP-BL subjects, respectively) was found in the group with partial blindness and retained tunnel vision, and functional connectivity between Broca’s area and the anterior calcarine sulcus (located at the periphery of the visual field) was increased in the RP-TV subjects compared to the sighted controls, suggesting that a partial sensory deficiency may be enough to induce functional plasticity. These findings of increasing functional connectivity may seem to contradict our findings of reduction in FA in the tracts of the ventral stream, as the earlier studies reported positive correlations between the strengths of functional and structural connectivities. However, the linkage between functional and structural connectivity is not perfect, and functional connectivity is thought to result from both mono- and polysynaptic circuits, and can be found between regions that are not linked structurally^43,44^. Moreover, since our study was focused on specific white matter tracts of the visual and motor systems, it does not rule out the possibility of a different pattern of change in other white matter tracts, for example those that are part of the language system, such as the superior longitudinal fasciculus. There is a need for further investigation to explore the relationships and temporal dynamics between the functional and structural plasticity processes that emerge during the decline in visual input.

The changes in diffusivity that we observed in a principal tract that is not part of the direct afferent route to the primary visual cortex further support the claim of preserved behavioral-related white matter plasticity in the adult brain^1,45,46^. In contrast to the direct elimination of visual input to the visual system, microstructural changes in motor system tracts, although demonstrated at a less stringent statistical threshold, point to behavioral and adaptive sources of plasticity following visual deprivation. Previous studies in late blind subjects have identified FA changes in the corticospinal tract, albeit with contradictory trends^7–9^. In a recent study of a group of patients with macular degeneration, no changes were found in this tract^14^. The trend we observed in the corticospinal tract of progressive reduction in FA and axial diffusivity from sight to partial vision to blindness might point to behaviorally-induced changes that coincide with studies showing the susceptibility of the corticospinal tract in the adult healthy brain to undergo structural modification following experience and training^47–49^.

Various neural components may affect the average measured diffusion in the voxel, hindering an exact biological interpretation of the changes in DTI indices. White matter plasticity may involve alterations in fiber organisation, density and axon diameter, as well as changes in the myelination of unmyelinated axons and modifications in myelin thickness. All of these can shape diffusivity along the tract^1^. Diffusion anisotropy in neural tracts was shown to relate to the axonal membrane, and to be modulated by myelin coverage^50^. In line with the interpretation of FA as a measure of axonal integrity^51^, the reduced anisotropy in neuronal tracts as a result of blindness has been attributed to axonal degeneration^7–10,25^. Studies examining the underlying axes of anisotropy (i.e. axial and radial diffusivities) have linked axial diffusivity to axonal integrity and radial diffusivity to myelination^52,53^. Increased axial diffusivity values were found to correlate with axolemma area^54^, axon counts and axon diameter^55^, whereas increases in radial diffusivity were attributed to a loss of myelin integrity^52,56^. Finally, glial components of the white matter play a role in plasticity processes and may also contribute to the changes observed in DTI. These structural modifications may include changes in astrocyte numbers and morphology, as well as proliferation and differentiation of oligodendrocyte precursor cells into myelinating cells^1^. Recent studies have shown that the electrical activity of axons promotes the proliferation and differentiation of oligodendrocyte precursor cells as well as of mature oligodendrocytes to form myelin^45,57^. Overall, therefore, different plasticity mechanisms, both those directly affected by the loss of neural input and those that are secondary to the damaged tissue and tract and may be attributed to behavioral adaptation, can contribute to tissue reorganisation and the observed changes in diffusivity.

In summary, in the present study we observed the gradual progression of white matter structural plasticity following partial to full obscuring of the visual field in two groups of subjects suffering from RP. Compared to the sighted controls we found an ongoing reduction in fiber integrity (indicated by changes in FA and radial diffusivity indices) in the optic tract and optic radiation, ranging from partial obscuring to full masking of the visual field. Our results also suggest that other central white matter tracts (such as the those of the ventral stream and the corticospinal tract) undergo gradually changing structural plasticity following reduction of the visual field, and that this may correspond to the behavioral adaptation and cross-modal plasticity subsequent to the sensory deprivation. The observed structural reorganisation in patients who still maintain some visual input may have clinical implications as to the timing of rehabilitative therapy.

## Methods

### Subjects

The sample comprised 32 participants assigned to three age-matched groups:

I. Ten subjects blinded by RP (RP-BL; 5 women; mean age ± SEM, 52.8 ± 3.9). Subjects had complete loss of the entire visual field, although some form of light perception might persist. Four were Braille readers.
II. Ten subjects with tunnel vision as a result of RP (RP-TV; 4 women; mean age ± SEM, 49.9 ± 4.4). The residual central visual field, as evaluated by Goldmann III/4 kinetic perimetry, was limited to a diameter of 10–20°. The best-corrected visual acuity (measured by EDTRS charts) was equal or superior to 20/40.
III. Twelve sighted controls (SC; 5 women; mean age ± SEM, 48.4 ± 4.6) with normal visual acuity and visual field (evaluated by Goldmann III/4 kinetic perimetry).

No subject had any reported neurological or psychiatric antecedents. The research protocol (#12873) was approved by the Ethics Committee (Comité de protection des personnes, Ile de France V, and Agence Nationale de Sécurité du Médicament et des Produits de Santé). All subjects gave their written informed consent. All methods were used in accordance with the relevant guidelines and regulations.

### Imaging data

Data were acquired on a 3 T clinical imager (Sigma Horizon) using an 8-channel head coil. The DTI protocol included whole-brain diffusion-weighted images with the following parameters: 1.25 × 1.25 × 2.5 mm^3^ resolution, 50 axial slices, Δ/δ 34.9/15.2 ms; phase-encoding direction, AP; b, 1000 s/mm^2^, 50 gradient directions, and one image with no diffusion weighting (b0). T1-weighted gradient-echo images were acquired with the following parameters: TE, 3.9 ms; TR,9.5 ms; flip angle, 20°; voxel size, 0.5 × 0.5 mm^2^ (FOV, 25.6 × 25.6 mm^2^; matrix, 512 × 512), slice thickness, 1.2 mm. Images were resampled to a resolution of 1 × 1 × 1.2 mm^3^ and skull-stripped in order to be applied as a subject-matched template in the EPI correction.

### DTI post-processing

DTI was analysed using ExploreDTI software^58^. Prior to DTI calculation the images were regularised using the ExploreDTI tool to de-noise the data. DTI was calculated by means of robust estimation^59^, and this was followed by correction for motion, EPI and susceptibility distortions using the matched T1-weighted images. Maps of mean diffusivity, fractional anisotropy (FA), radial diffusivity (RD) and axial diffusivity were produced.

Before processing, two subjects were excluded (one from the RP-BL group and one from the SC group) because of technical problems with their matching T1-weighted maps. Two other subjects (one from the RP-BL and one from the SC group) were removed from further analysis because of severe EPI artifacts.

### Tract-Based Spatial Statistics

For voxel-based white matter analysis we used Tract-Based Spatial Statistics (TBSS)^24^, which is part of the FSL software package^60^. Briefly, nonlinear registration transformed all FA images into a standard space using a template specific to this study. To create this study-specific template, all FA images were co-registered using nonlinear registration. The FA image with the smallest average warping was chosen as the target image and was affine-aligned into an MNI152 standard space. The non-linearly transformed FA images were averaged to create a mean FA image, which was then thinned at a threshold of FA > 0.2 to create a mean white matter skeleton. Local FA maxima from the aligned FA images of each subject were then projected onto the mean FA skeleton. The same spatial transformations were applied on the mean diffusivity, axial diffusivity and radial diffusivity maps of each participant, as well as on their matching tract masks (see below).

### Tractography

Since we focused here on specific tracts of the visual pathway (optic tract, optic radiation, IFOF, ILF) and the corticospinal tract of the motor system, we restricted our analyses in the computed group skeleton to these main pathways using tract masks^61–63^. To delineate these pathways we performed deterministic streamline tractography using constrained spherical deconvolution (CSD)^64,65^. The fiber orientation distribution functions (FOD) were estimated with CSD, and we carried out whole brain tractography with the following tracking parameters: seed point resolution in each voxel set at 1 × 1 × 1.2 mm^3^; step size, 0.5; streamline turning angle threshold, 30°; and tract length of 50–500 mm.

Based on anatomical atlases^23,66^ and previous reports^10,67–70^, we developed a protocol for semi-automatic extraction^71^ of the tracts. The protocol for reproducible extraction of the tracts was based on the results obtained in a blind subject using the “AND” and “NOT” drawing tool in ExploreDTI. Each of this subject’s tracts was manually corrected for outlier streamlines, exported as maps, and then spatially transformed using the same spatial transformation as that calculated for the TBSS pipeline.

### Statistics

Statistical analyses were restricted to the skeleton voxels that are part of the main visual pathway (optic tract, optic radiation), the ventral visual tracts (ILF and IFOF) and the corticospinal tract (CST), by the use of masks constructed from the tractography analyses. These tract masks were made by merging the spatially transformed tract maps that were obtained from all subjects and consisted of voxels with at least 60% overlap between subjects.

A contrast of interest was applied to determine the difference in diffusion indices (FA, mean diffusivity, radial diffusivity and axial diffusivity) and to test for a linear trend of change in diffusivity indices from SC to RP-TV to RP-BL, when controlling for the age of the subjects. In the RP-tunnel-vision group, we carried out partial correlation analyses between the diffusion indices, the mean visual acuity (average of left and right visual acuity scores shown in Table 1) and the duration of visual deficit, while controlling for age. A visual-deficit duration record was missing for one RP-TV subject, and this correlation analysis was therefore run on nine of the ten subjects. Non-parametric voxel-based permutation tests (5000 permutations) were conducted using the Randomise package (part of FSL)^72^. Test statistics were corrected for multiple comparisons using the Threshold-Free-Cluster-Enhancement (TFCE)^73^ and a threshold of p < 0.05 FWE.

**Table 1:** Subjects’ clinical data. RP-BL, RP blind subjects; RP-TV, RP tunnel vision subjects; SC: sighted controls. Blindness duration, number of years since the subjects lost any form vision in all parts of their visual field but might still retain some bare light perception.

Group	Subject	Age	Sex	Dominant hand	Braille	Blindness duration	Visual deficit duration	Left eye visual acuity (logmar)	Right eye visual acuity (logmar)
RP-BL	1	60	F	R	yes	15		None	None
	2	62	F	R	no	6		None	None
	3	44	M	R	no	14		None	None
	4	47	F	R	no	12		None	None
	5	57	F	R	yes	17		None	None
	6	59	M	R	no	6		None	None
	7	62	M	R	yes	19		None	None
	8	31	F	R	yes	10		None	None
RP-TV	1	43	F	L			15	0.1	0.1
	2	54	M	R			33	0	0.1
	3	62	M	R			23	0.1	0.1
	4	37	F	R			17	0.3	0.3
	5	28	F	R			12	0	0
	6	60	M	R/L			56	0.3	0.2
	7	61	F	R			46	0.3	0.2
	8	59	M	R			53	0.3	0.3
	9	63	M	R			N/A	0.5	0.6
	10	29	M	R			19	0.4	0.2
SC	1	31	M	R					
	2	28	F	R					
	3	59	F	R					
	4	59	M	R					
	5	42	F	R					
	6	61	F	R					
	7	28	F	L					
	8	56	M	R					
	9	63	F	R					
	10	57	M	R					

Visual deficit duration, age minus age at disease onset.

## Data Availability

The dataset analysed during the current study is available from the corresponding author on reasonable request.
