# Author Correction: The development of white matter structural changes during the process of deterioration of the visual field

**DOI:** 10.1038/s41598-021-89837-6

**Published:** 2021-05-20

**Authors:** Shir Hofstetter, Norman Sabbah, Saddek Mohand-Saïd, José-Alain Sahel, Christophe Habas, Avinoam B. Safran, Amir Amedi

**Affiliations:** 1 Department of Medical Neurobiology, The Institute for Medical Research Israel–Canada, Faculty of Medicine, The Hebrew University of Jerusalem, 91220 Jerusalem, Israel; 2 The Edmond and Lily Safra Center for Brain Sciences, The Hebrew University of Jerusalem, 91220 Jerusalem, Israel; 3 Sorbonne Université, INSERM, CNRS, Institut de la Vision, F-75012 Paris, France; 4 CHNO des Quinze-Vingts, DHU Sight Restore, INSERM-DGOS CIC 1423, F-75012 Paris, France; 5 Fondation Ophtalmologique A. de Rothschild, F-75019 Paris, France; 6 Department of Ophthalmology, University of Pittsburgh School of Medicine, Pittsburgh, PA 15213 USA; 7 Department of Clinical Neurosciences, Geneva University School of Medicine, Geneva, Switzerland; 8 Centre de Neuro-Imagerie, Centre Hospitalier National d’Ophtalmologie des Quinze-Vingts, F-75012 Paris, France; 9 The Cognitive Science Program, The Hebrew University of Jerusalem, 91220 Jerusalem, Israel

Correction to: *Scientific Reports* 10.1038/s41598-018-38430-5, published online 14 February 2019

The Acknowledgements section in the original version of this Article was incomplete.

“This work was supported by the French State programme “Investissements d’Avenir” managed by the Agence Nationale de la Recherche [LaBex LIFESENSES: ANR-10-LABX-65] and Groupe Optic 2000.”

now reads:

“This work was supported by the French State programme “Investissements d’Avenir” managed by the Agence Nationale de la Recherche [LaBex LIFESENSES: ANR-10-LABX-65] and Groupe Optic 2000, the ERC Consolidator Grant (773121), a James S. McDonnell Foundation, United States, scholar award (no. 652 220020284), and a Joy ventures grant to A.A.”

The original Article has been corrected.

